# Rapid, sensitive, and specific detection of SARS-CoV-2 in nasopharyngeal swab samples of suspected patients using a novel one-step loop-mediated isothermal amplification (one-step LAMP) technique

**DOI:** 10.1186/s12866-023-02806-z

**Published:** 2023-03-07

**Authors:** Sayyad Khanizadeh, Asra Malekshahi, Hooman Hanifehpour, Mehdi Birjandi, Shirzad Fallahi

**Affiliations:** 1 Hepatitis Research Center, School of Medicine, Lorestan University of Medical Sciences, Khorramabad, Iran; 2 Department of Virology, School of Medicine, Lorestan University of Medical Sciences, Khorramabad, Iran; 3 Department of Microbiology, Cancer Biomedical Research Center (CBC), Tehran, Iran; 4 Department of Biostatistics and Epidemiology, School of Health and Nutrition, Lorestan University of Medical Sciences, Khorramabad, Iran; 5 Department of Parasitology and Mycology, School of Medicine, Lorestan University of Medical Sciences, Khorramabad, Iran

**Keywords:** Rapid, Sensitive, Detection, SARS-CoV-2, One-step LAMP, One-step RT-qPCR

## Abstract

**Background:**

In the absence of effective antiviral drugs or vaccines, early and accurate detection of SARS-CoV-2 infection is essential to the COVID-19 pandemic. This study developed and evaluated a novel rapid One-Step LAMP assay to directly detect the SARS-CoV-2 RNA from nasopharyngeal (NP) swab samples of patients with suspected SARS-CoV-2 infection living in deprived areas in comparison to One-Step Real-time PCR.

**Methods:**

Two hundred fifty-four NP swab samples from patients suspected of COVID-19 infection living in deprived western areas of Iran were tested by TaqMan One-Step RT-qPCR and fast One-Step LAMP assays. Tenfold serial dilutions of SARS-CoV-2 RNA standard strain where the viral copy number in each dilution was previously determined using the qPCR and various templates were used to investigate the analytical sensitivity and specificity of the One-Step LAMP assay in triplicate. Also, the efficacy and reliability of the method compared to TaqMan One-Step RT-qPCR were evaluated using SARS-CoV-2 positive and negative clinical samples.

**Results:**

The results of the One-Step RT-qPCR and One-Step LAMP tests were positive in 131 (51.6%) and 127 (50%) participants, respectively. Based on Cohen’s kappa coefficient (κ), the agreement between the two tests was 97%, which was statistically significant (*P* < 0.001). The detection limit for the One-Step LAMP assay was 1 × 10^1^ copies of standard SARS-CoV-2 RNA per reaction in less than an hour in triplicates. Negative results in all samples with non-SARS-CoV-2 templates represent 100% specificity.

**Conclusions:**

The results showed that the One-Step LAMP assay is an efficient consistent technique for detecting SARS-CoV-2 among suspected individuals due to its simplicity, speed, low cost, sensitivity, and specificity. Therefore, it has great potential as a useful diagnostic tool for disease epidemic control, timely treatment, and public health protection, especially in poor and underdeveloped countries.

**Supplementary Information:**

The online version contains supplementary material available at 10.1186/s12866-023-02806-z.

## Background

Since December 2019, COVID-19, an infectious disease caused by the (Severe Acute Respiratory Syndrome Coronavirus 2) SARS-CoV-2 coronavirus has emerged in Wuhan, China [1, 2]. SARS-CoV-2 has a stronger human-to-human transmission capacity than SARS-CoV and MERS-CoV [3]. By April 30, 2021, the virus had infected 93,603,141 and killed 2,004,041 people in the world, causing a serious crisis worldwide [2]. In the absence of effective antiviral drugs or effective vaccines, early detection of SARS-CoV-2 infection is essential to restrain COVID-19, especially in poor or undeveloped countries [4, 5]. Extensive molecular diagnostic testing for the virus is crucial for rapid diagnosis, quarantine, treatment, and managed response to the SARS-CoV-2 pandemic [6], especially if there are many asymptomatic cases with the frequency seen in recent pandemics [7]. Since the outbreak of COVID-19, several Real-time PCR (RT-qPCR) methods have been developed and play a key role in the laboratory confirmation of SARS-CoV-2 infection [2, 8, 9]. Although RT-qPCR methods are used as the routine standard method in diagnosing pathogens due to their high sensitivity and specificity, there are still many concerns. In summary, high-level laboratory facilities, expensive and sophisticated equipment which is the main problem of countries with poor economic conditions, trained personnel, and upwards of 4–8 h to process are required to perform RT-qPCR tests correctly [10]. Due to the time-consuming nature of this technique, limits its capacity to respond to the demand for virus diagnosis in a rapidly growing number of patients with COVID-19 infection, suspected of infection, or close contact with confirmed cases [11]. Therefore, an alternative, rapid, simple, and sensitive care test is needed to facilitate the diagnosis of SARS-CoV-2 infection in areas with limited facilities [5, 12]. To overcome the limitations of RT-qPCR and to identify the nucleic acids of pathogens with high sensitivity and specificity, isothermal amplification methods such as the Loop-mediated isothermal amplification (LAMP) technique have been developed. LAMP was first developed by Notomi et al. in 2000 [13]. This test is a simple, fast, sensitive, and effective method for amplifying and detecting nucleic acids [14]. In the LAMP technique, a set of four (or six) primers binds to six (or eight) different regions of the target gene, making this technique specifically unique [13]. This primer set consists of two external primers (F3 and B3), two internal primers (internal forward primer (FIP) and internal reverse primer (BIP)), and two loop primers (forward loop and backward loop). The LAMP reaction can be easily performed under Isothermal conditions [15] using the *Bst DNA polymerase* enzyme which has high strand displacing activity [13]. The *Bst DNA polymerase* used in LAMP is more resistant than that used in traditional PCR and can therefore be resistant in the presence of PCR inhibitors that are often found in body fluids such as saliva and viral transport media (VTM) [16–18]. It also works in the presence of RNA [19]. The LAMP method can also amplify many copies of DNA in less than an hour and does not require a specific reagent, or expensive and sophisticated equipment. Thus, it can be used in field conditions as well as in countries with poor financial resources. This method can be used for both DNA and RNA purposes. Because it can detect the target RNA by using reverse transcriptase (RT-LAMP) [20]. LAMP is currently used to identify a wide range of pathogens, including positive-sense RNA viruses in humans [21–23]. The studies conducted on the LAMP technique in diagnosing SARS-CoV-2 worldwide have shown remarkable and promising results [10, 11, 14, 22, 24–37].

In this study, a rapid One-Step LAMP method targeting the N gene of the SARS-CoV-2 virus is developed and applied for the diagnosis of SARS-CoV-2 in nasopharyngeal (NP) swap sample of patients suspected of COVID-19 from one of the western regions of Iran with poor socio-economic conditions. Also, the efficacy, reliability, and consistency of the results of this method were evaluated compared to TaqMan One-Step RT-qPCR using positive and negative clinical samples of COVID-19.

## Methods

### Patients and samples

A total of 254 NP swab samples from patients suspected of SARS-CoV-2 infection were collected in 2 ml of fresh viral transport media (VTM) after completing the questionnaire and obtaining written consent at the Hepatitis Research Center, the reference center for SARS-CoV-2 diagnosis in Lorestan Province, Khorramabad, deprived western areas of Iran for 9 months coinciding with the rise of the COVID-19 outbreak in Iran as well as in Khorramabad City. According to the manufacturer, instructions, the RNA was extracted from NP samples using an RNA extraction kit (COVID-19 RNA Extraction Kit, Biorexfars Co. Iran). The RNA extraction procedure briefly includes 4 steps; a) lysing the samples, b) attachment of the RNA to the filter membrane, c) filter washing and alcohol removal, and lastly d) separation of RNA from the filter. Firstly, the samples were lysed at 37 ͦ С in the presence of Lysis Buffer and Proteinase K. After the samples were lysed, the RNA is attached to the filter, and during the washing process, other additional substances are removed, and the purified RNA is dissolved in the Elution Buffer. Residual contaminants are washed away using Wash Buffer 1 and 2, while the nucleic acids remain attached to the filter membrane. High-purity viral RNA is removed from the filter membrane using Elution Buffer. The separation process was done with 100 μl of Elution Buffer. Using a small volume of Elution Buffer solution can increase the concentration of RNA. NanoDrop OneC equipment (NanoDrop Technologies Inc., Wilmington, DE by Thermo Scientific, USA) was used to measure the RNA concentrations and purity. Based on the recommended guidelines, when the ratio of 260/280 was ~ 2.0 the RNA purity was considered adequate and acceptable. The harvested RNA samples were kept at − 70 °C until the next molecular valuations.

### Ethics approval and consent to participate

The Ethical Committee of the Vice-Chancellor of Research and Development of the Lorestan University of Medical Sciences, Khorramabad, West of Iran, reviewed and approved the study protocols and experiments under the registration number IR.LUMS.REC.1399.058. The written informed consent of all study participants or their legal representatives, especially in the case of critically ill patients was obtained for this study. As well as, the authors confirm that all methods and experiments were performed by relevant guidelines and regulations.

### One-step RT-qPCR

**Fig. 1:**
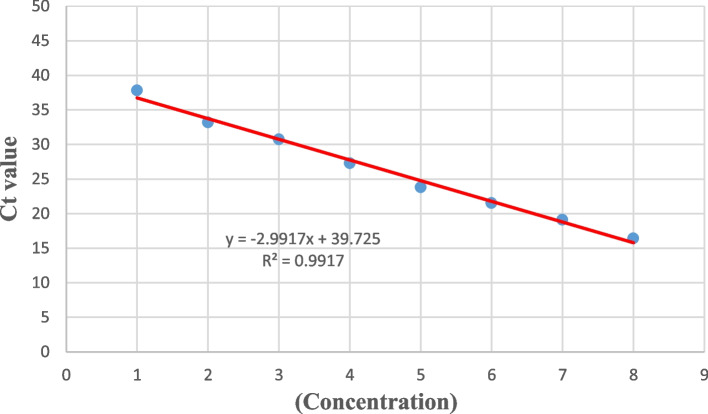
Calibration curves of TaqMan One-step RT-qPCR. Amplification and analyzing the serially diluted RNA targets in the One-step RT-qPCR protocol. The Ct (threshold cycle) values were plotted against the concentration of RNA standards. The linear regression curve (y) and the coefficient of determination (R2) were calculated for everyone

The COVID-19 TaqMan One-Step RT-qPCR Kit (Pishtaz Teb Co., Iran/ PT. COVID.100) was used for routine tests of COVID-19 among the suspected patients. The primer-probe of this kit adopts the dual-target gene design, which targets the specific conserved sequence encoding the RdRp (RNA-dependent RNA polymerase) and the nucleocapsid protein N regions of SARS-CoV-2 (GenBank MN908947, Wuhan-Hu-1). The TaqMan One-Step RT-qPCR assay was performed in a 20 μl reaction volume containing 5 μl RNA template, 9 μl resuspended master mix, 2 μl N/ICON Primer & probe mix (HEX/ROX), and 5 μl RNase-free water was prepared. After adding the reagents to the tubes, the lids were covered immediately, spin down briefly using a centrifuge to remove air bubbles, and then transferred to the amplification area. The PCR machine used in this research was the Rotor-Gene Q-Pure Detection Real-Time PCR system (Rotor-Gene Q MDx – QIAGEN, USA). To detect SARS-CoV-2 RNA, the HEX (N gene) and ROX (RdRp) channels were selected for internal control. To perform the reverse transcription reaction 20 minutes at 50 °C cycles were conducted, the initial cDNA denaturation was performed at 95 °C for 3 minutes, followed by 45 cycles of denaturation at 95 °C for 15 s and annealing, extension, and fluorescence measurement at 55° for the 40s, and a final cooling at 25 C for 10s (Fig. 1).

### One-step LAMP

**Fig. 2:**
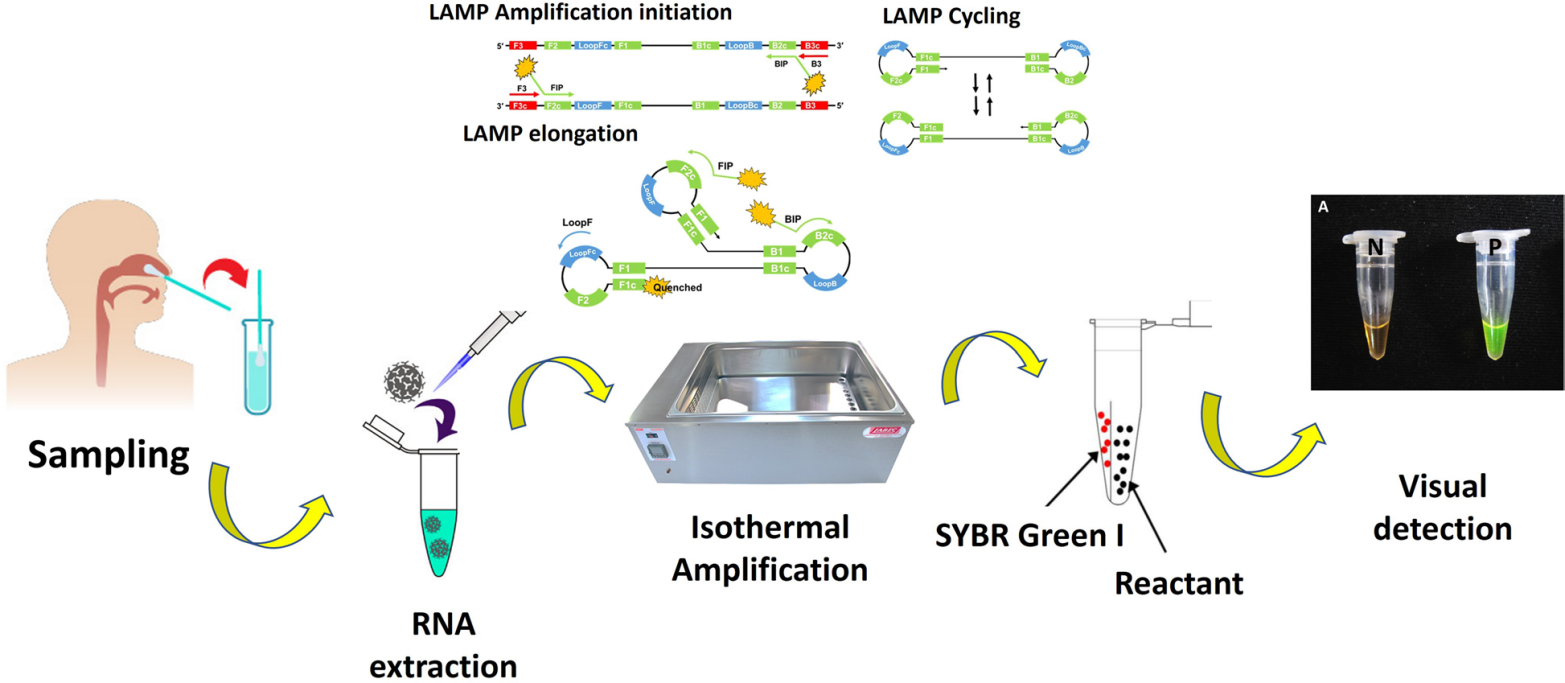
Schematic image of the detection of SARS-CoV-2 in nasopharyngeal swab samples of suspected patients using the One-Step LAMP technique. P, Positive control; N, Negative control

The One-Step LAMP assay was performed in a 25 μl reaction mixture containing 5 pmol each of F3 and B3 external primers, 40 pmol each of FIP and BIP internal primers, 20 pmol each of LF and LB loop primers (Table 1) [24] which targeted the N gene of SARS-CoV-2 (GenBank MN908947, Wuhan-Hu-1), 8 U (1 μl) of *Bst DNA Polymerase 3.0* (New England Biolabs, USA), a new version of the key enzyme of the LAMP reaction, in 2.5 μl of isothermal amplification buffer [20 mM Tris–HCl, 10 mM (NH4)_2_SO_4_, 150 mM KCl, 2 mM MgSO_4_, 0.1% Tween® 20 (pH 8.8 @ 25 °C)], 8 mM MgSO_4_, 0.8 M betaine (Sigma-Aldrich), 1.4 mM deoxynucleoside triphosphates (dNTP), and 1 μl of template RNA. The genomic RNA of standard SARS-CoV-2 strain (229E) and double deionized distilled water were used as positive and negative controls in each run, respectively. The reaction mixture was incubated at 64 °C for 30 min using a water bath, afterwards, the reaction was inactivated by a 2 min incubation at 80 °C. Toward the end of visual detection of the resulting amplicons, 3 μl of 1:10 diluted 10,000 × concentration SYBR Green I (Invitrogen Carlsbad, CA, USA) was added to the reaction tubes. Given that opening LAMP tubes after the reaction is generally considered bad practice and is best avoided as it produces the risk of carryover contamination, after preparing the reaction mixture of the LAMP and allocating it between the microtubes and before incubating at 64 ͦ С, CybrGreen dye which normally is added to the microtubes at the end of the reaction when the incubation time is over and the template is highly amplified, was applied to the lid of the tube without getting into contact with the reaction liquid, and then the microtubes were gently placed in a water bath so that the CybrGreen dye remained attached to the inner surface of the microtube lid. After the reaction is finished, the attached dye is poured from the lid into the reaction mixture with a quick spin, and the test result can be read without opening the microtubes lid [37]. In a positive LAMP sample, green fluorescence was observed, while in the negative one, it remained the original pinkish-orange (Fig. 2). Preventive measures were taken to avoid carryover contamination between reactions. To confirm the results, after the LAMP tests of all the samples were done, 10 μl of the LAMP products was electrophoresis on a 1.5% agarose gel stained with DNA-safe stain (Nedayefan Co., Iran).

**Table 1:** Characteristics of primers used for the One-Step LAMP technique [24]

Target gene	Primer sequence	Primer length (nucleotide)
N (Nucleocapsid protein)	**F3 5′-CTACCTAGGAACTGGGCC-3’**	**18**
	**B3 5′-AGAAGAGGCTTGACTGCC-3’**	**18**
	**FIP 5′-GGTGTATTCAAGGCTCCCTCACCTATGGTGCTAACAAAGAC-3’**	**41**
	**BIP 5′-AATCCTGCTAACAATGCTGCAATCCTGCTCCCTTCTGCGTAG-3’**	**42**
	**LF 5′-GTTGCAACCCATATGATGC-3’**	**19**
	**LB 5′-CTTCCTCAAGGAACAACAT-3’**	**19**

### Analytical sensitivity and specificity of the one-step LAMP assay

To determine the analytical sensitivity of the One-Step LAMP assay, tenfold serial dilutions from 1× 10^6^ to 1 × 10^− 3^ copies of the RNA standard strain of SARS-CoV-2 were prepared in 1X HBSS (Gibco, 14,025–092) using the qPCR. The copy numbers of the RNA standard in each dilution were calculated using the qPCR according to Ji and colleagues’ method [38]. The accuracy of the analytical sensitivity results was confirmed by repeating the tests three times. Also, to ensure the results obtained for the analytical sensitivity test and to avoid possible visual error in the reaction tubes’ color examination, the reaction product was electrophoresed on a 1.5% agarose gel and evaluated under UV in the gel documentation. A set of 15 positive and 10 negative clinical samples previously tested by RT-qPCR were also selected to determine the clinical sensitivity of the One-Step LAMP assay using the optimized One-Step LAMP protocol.

The analytical specificity of the One-Step LAMP assay was examined by detecting the various templates, including *Influenza A virus*, *Influenza B virus*, *Respiratory syncytial virus*, *Adenovirus*, *Parainfluenza virus*, *Klebsiella pneumoniae*, *Streptococcus pneumoniae*, *Haemophilus influenza*, *Pseudomonas aeruginosa*, *Legionella pneumophila*, *Bordetella Pertussis*, *Staphylococcus aureus*, *Mycoplasma pneumoniae*, and *Chlamydia pneumoniae* as well as human positive samples of HIV ، HBV ، HCV ، EBV ، CMV ، HPV, and HSV1, and 2., and synthetic nucleic acid sequences prepared as a gift from Infectious Disease Control Center, Ministry of Health and Medical Education, Tehran, Iran.

### Statistical analysis

The data were analyzed using the SPSS 22 for windows (SPSS Inc., Chicago, IL, USA). The frequency distribution tables were used to describe the data. The probable significant statistical relationship between the variables was examined by Chi-square (χ2) and Fisher exact statistical tests and Cohen’s kappa coefficient (κ) calculates the agreement of the molecular tests. Multivariate modeling of data was performed using logistic regression. After adjustments, associations were tested using odd ratios (OR) and 95% confidence intervals (CI). At expected frequencies of less than five, the statistical significance was calculated using the Monte Carlo method simulation based on 10,000 replicates. The statistically significant level in all tests was considered 0.05.

## Results

### Demographic characteristics of participants

In the current study, 254 patients suspected of SARS-CoV-2 infection referred to the Hepatitis Research Center of Lorestan University of Medical Sciences, COVID-19 Diagnosis Reference Center in Khorramabad, deprived western areas of Iran, were included. Out of 254 participants, 132 people (52%) were male and 137 people (53.9%) lived in urban areas. The mean age of participants in the study was 37.68 ± 17.23 years, ranging from 1 year to 85 years. The most common clinical symptoms reported among the participants were muscle aches (28.7%), headache (27.2%), and sore throat (25.6%), respectively. While 2.4% of the participants in the study did not report any clinical manifestations. Computed tomography (CT) scan of the lungs showed lung involvement in 28 participants (11%), and 27 participants (10.6%) had at least one chronic UHC. The most chronic UHC reported among participants in the study were endocrine diseases (4.3%), and cardiovascular diseases (2.8%) respectively (Table 2).

**Table 2:** Demographic characteristics of suspected patients of SARS-COV-2 participating in the study

Variables	Groups	n (%)
Gender	Male	132 (52.00)
	Female	122 (48.00)
Occupancy	Urban	137 (53.90)
	Rural	117 (46.10)
Clinical symptoms	Fever	23 (9.10)
	Fever & Chills	54 (21.30)
	Muscle ache	73 (28.70)
	Headache	69 (27.20)
	Sore throat	65 (25.60)
	Cough	52 (20.50)
	Loss of smell & taste	21 (8.30)
	Diarrhea & Vomiting	5 (2.0)
	Shortness of breath	27 (10.60)
	Asymptomatic	6 (2.40)
	Other	31 (12.20)
Underlying health conditions (UHC)	Endocrine diseases	11 (4.30)
	Liver diseases	2 (0.80)
	Kidney diseases	5 (2.00)
	Gastrointestinal diseases	2 (0.80)
	Pulmonary diseases	4 (1.6)
	Neurological diseases	0 (0.0)
	Cardiovascular diseases	7 (2.80)
	Cancer disease	1 (0.40)
	No UHC	227 (89.37)
Lung involvement in CT scan (LICT)	Positive	28 (11.00)
	Negative	226 (89.00)
Age category	≤ 18	35 (13.80)
	19–64	199 (78.30)
	≥ 65	20 (7.90)
Age (Mean ± SD)		37.68 ± 17.23

### One-step RT-qPCR and one-step LAMP

**Fig. 3:**
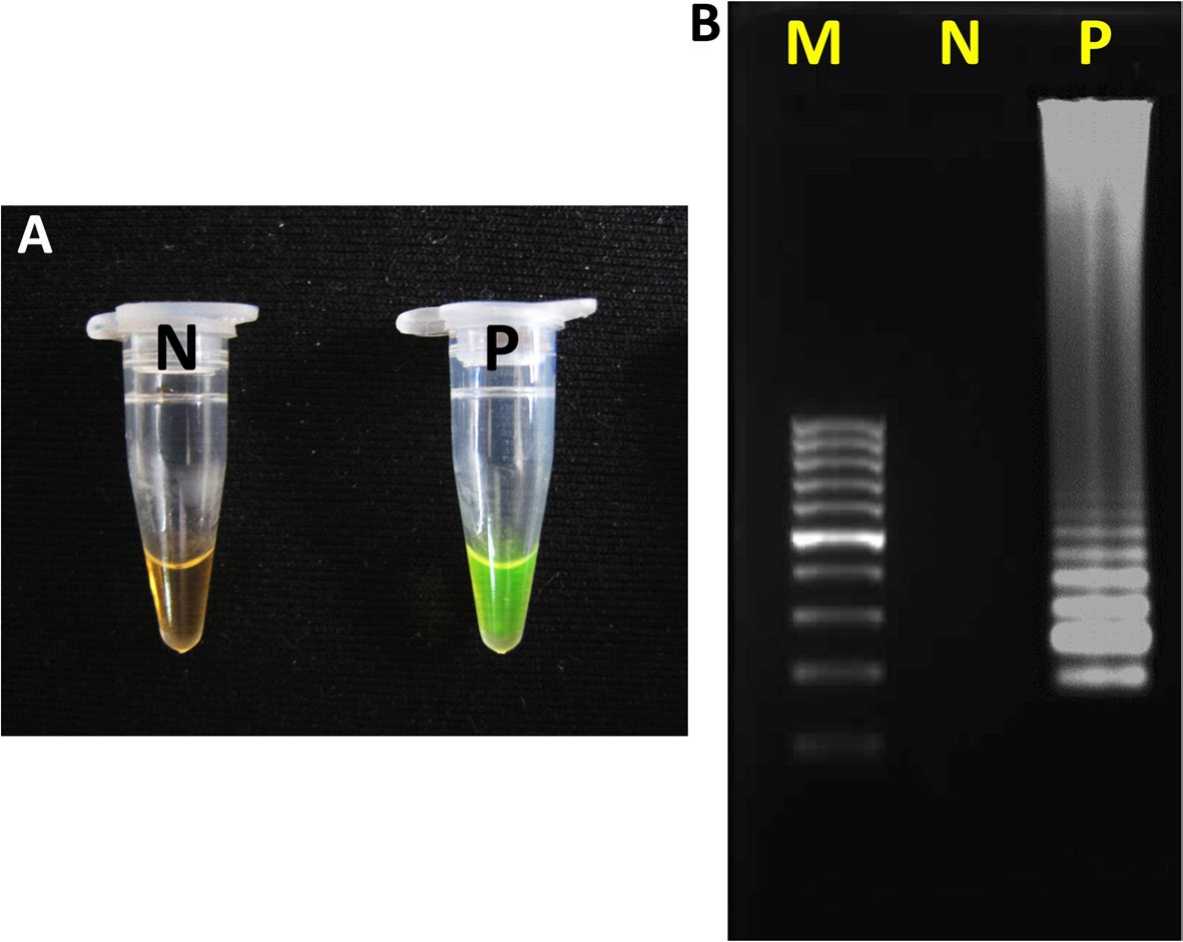
Monitoring of LAMP amplification of the SARS-CoV-2 RNA. **A** Visual inspection of the RNA amplification by fluorescence of the reaction mixture under normal light. **B** Agarose gel analysis of LAMP amplified product. P, Positive control; N, Negative control; M, 100 bp molecular weight marker

Regarding SARS-CoV-2 infection, the result of TaqMan One-Step RT-qPCR and One-Step LAMP assays were positive in 131 (51.6%) and 127 (50%) of the participants, respectively. Based on Cohen’s kappa coefficient (κ), the agreement rate between the two tests was 97%, which considering *P* < 0.001 was statistically significant (Table 3). According to the manufacturer’s declaration, the primers and probes provided in the RT-qPCR kit were designed based on the conserved sequence of the novel coronavirus (SARS-CoV-2), and have a high detection rate of the target gene fragment. Also, the RT-qPCR kit had no cross-reactions among positive samples of coronavirus (NL63, HKU1, 229E, OC43). The One-Step LAMP assay was performed in a 25 μl reaction mixture using the new version of the key enzyme of the LAMP reaction, *Bst DNA Polymerase 3.0* (New England Biolabs, USA) (Fig. 3A and B).

**Table 3:** The consensus of the One-Step RT-qPCR test and One-Step LAMP technique results in the diagnosis of SARS-CoV-2

		One-Step RT-qPCR		Total	*P* < 0.001
		Negative	Positive		
		n (%)	n (%)		
**One-Step LAMP**	Negative	123 (48.4)	4 (1.6)	127 (50.0)	Kappa = 97%
	Positive	0 (0.0)	127 (50.0)	127 (50.0)	
	Total	123 (48.4)	131 (51.6)	254 (100.0)

### Analytical sensitivity and specificity of the one-step LAMP assay

**Fig. 4:**
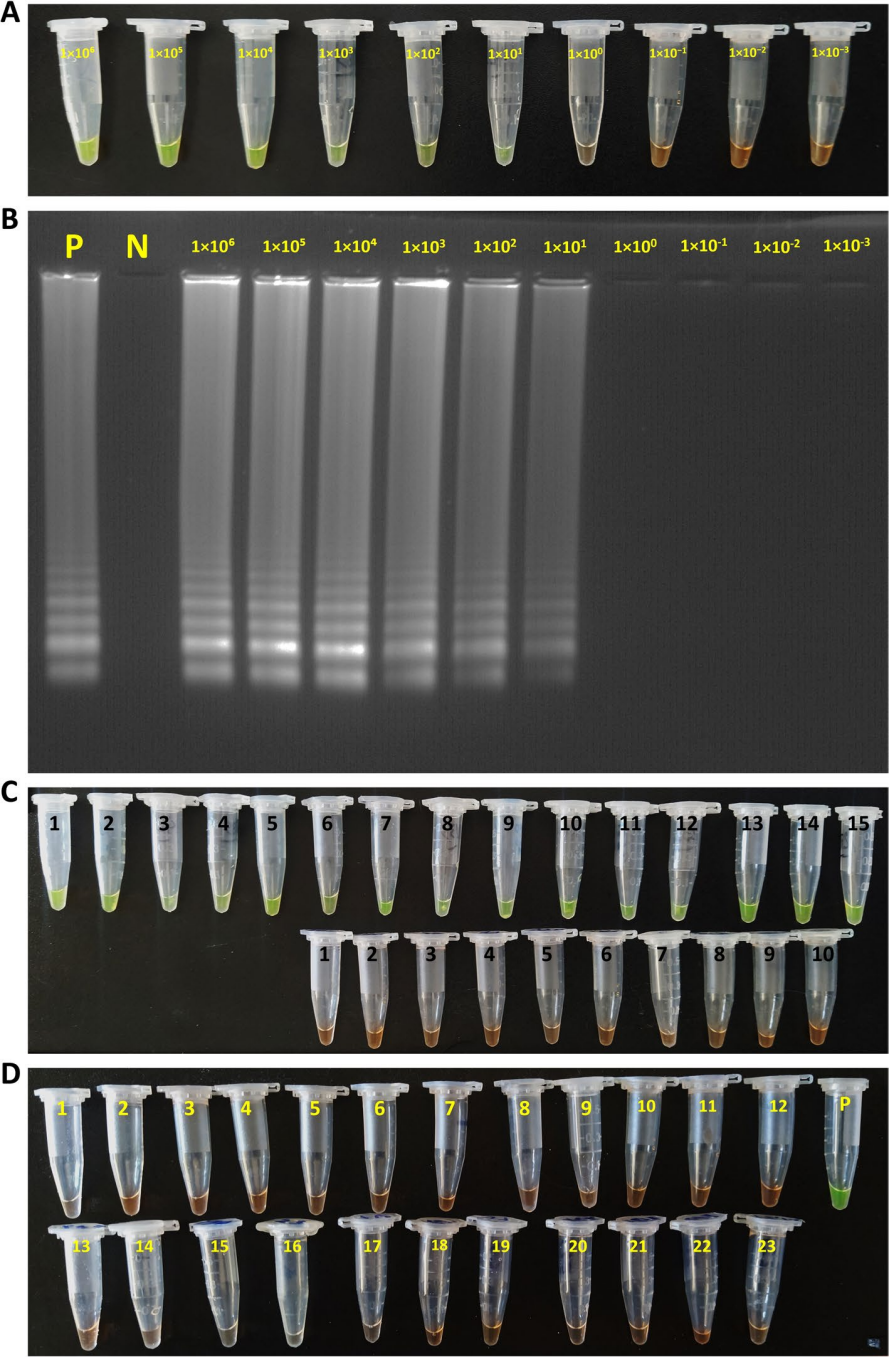
Analytical and clinical sensitivity and specificity of the One-step LAMP assay for the detection of SARS-CoV-2 RNA based on the N gene amplification. **A** and **B** tenfold serial dilutions of the RNA standard of SARS-CoV-2 from 1 × 10^6^ to 1 × 10^− 3^ copies per reaction. **C** Positive and negative SARS-CoV-2 clinical samples from participants in the study. **D** Various non-SARS-CoV-2 templates, including Influenza A virus, Influenza B virus, Respiratory syncytial virus, Adenovirus, Parainfluenza virus, *Klebsiella pneumoniae*, *Streptococcus pneumoniae*, *Haemophilus influenza*, *Pseudomonas aeruginosa*, *Legionella pneumophila*, *Bordetella Pertussis*, *Staphylococcus aureus*, *Mycoplasma pneumoniae*, and *Chlamydia pneumoniae* as well as human positive samples of HIV ، HBV ، HCV ، EBV ، CMV ، HPV, and HSV1, and 2., and synthetic nucleic acid sequences for specificity assessment of the LAMP technique. P, Positive control

To determine the analytical sensitivity of the One-Step LAMP assay tenfold serial dilutions of the RNA standard of SARS-CoV-2 from 1 × 10^6^ to 1× 10^− 3^ copies were used. The limit of detection for the One-Step LAMP assay was 1 × 10^1^ copies of the RNA standard of SARS-CoV-2 per reaction in all three repeats in less than an hour. Electrophoresis of the LAMP reaction products on 1.5% agarose gel and examination in a gel documentation system also reaffirmed the results (Fig. 4A and B). Moreover, all patients’ positive and negative clinical samples had the same positive and negative results using the optimized One-Step LAMP protocol. The positive results observed in all positive controls and a negative result in all samples with non-SARS-CoV-2 templates represent 100% specificity of the One-Step LAMP assay. In these samples, the only unchanged pale-orange color of the CybrGreen I observed indicates no cross-reactivity with non-SARS-CoV-2 templates (Fig. 4C and D). Due to the limited access, no specificity test was performed for *Enterovirus*, *Chlamydia pneumonia*, *Mycobacterium tuberculosis*, human *Metapneumovirus*, *Streptococcus pyrogenes*, *Pneumocystis jirovecii*, *Candida albicans*, *SARS-coronavirus*, and *Mers-Coronavirus*.

### The probable relationship

Given that currently, the most common and accurate test for diagnosing SARS-CoV-2 infection among suspects is the Real-time PCR test, the relationship between variables and infection with SARS-CoV-2 was evaluated based on the results of the One-Step RT-qPCR assay. More than half of the patients (52.3%) with a positive test for SARS-CoV-2 by TaqMan One-Step RT-qPCR assay, were men and the rest were women. That at *P* > 0.05, there was no statistically significant relationship between gender and COVID-19 infection. The mean age of individuals with positive One-Step RT-qPCR test results in terms of COVID-19 infection was 38.32 ± 16.95 and the mean age of individuals with negative One-Step RT-qPCR test results was 37 ± 17.57, respectively which according to *P* = 0.54 there was no statistical difference between the mean age of the two groups. Ninety (65.7%) of people with a positive result for COVID-19 infection using the One-Step RT-qPCR assay were residents in urban areas and the rest were residents of rural areas, according to *P* < 0.001, a statistically significant relationship was found between the residency and infection with SARS-CoV-2. The most common clinical complications reported by patients with SARS-CoV-2 were muscle aches (~33%), sore throat (~31%), and headache (~28.5%), respectively, and the least reported clinical symptoms were diarrhea and vomiting (3.1%). Six patients (4.7%) with positive One-Step RT-qPCR and One-step LAMP test results reported no clinical signs. Statistical analysis of the data using the Chi-square test showed that there was a statistically significant relationship between infection with SARS-CoV-2 using the One-Step RT-qPCR test and clinical signs of fever and chills (*P* = 0.03), and shortness of breath (*P* < 0.001) while there was no statistically significant relationship between clinical signs of the fever (*P* = 0.95), headache (*P* = 0.49), muscle aches (*P* = 0.13), cough (*P* = 0.10), diarrhea and vomiting (*P* = 0.23), and infection with COVID-19. The statistical relationship between coronavirus infection using the TaqMan One-Step RT-qPCR test and clinical signs of sore throat (*P* = 0.06) and loss of taste and smell (*P* = 0.06) was remarkable but insignificant.

Statistical analysis of data using the Chi-square test showed that there is a statistically significant relationship between the results of the TaqMan One-Step RT-qPCR test in terms of SARS-CoV-2 infection, and chronic UHC (*P* = 0.006) so 21 patients with a positive One-Step RT-qPCR result had at least one UHC. Moreover, statistical data analysis using the Chi-square test showed no statistically significant relationship between Covid-19 infection using the TaqMan One-Step RT-qPCR and lung, liver, kidney, gastrointestinal, cardiovascular, and cancer UHC (*P* > 0.05). Also, the relationship between SARS-CoV-2 infection and endocrine diseases was insignificant but remarkable (*P* = 0.06) (Table 4).

**Table 4:** Univariate relationship of the SARS-CoV-2 infection using One-Step RT-qPCR assay and One-Step LAMP technique with demographic variables

Variables		One-Step RT-qPCR		***P***-value	Odds Ratio (CI %95)	One-Step LAMP		***P***-value	Odds Ratio (CI %95)
		Negative	Positive			Negative	Positive		
		n (%)	n (%)			n (%)	n (%)		
**Gender**	**Male**	63 (47.7)	69 (52.3)	0.817	1.060 (0.648–1.735)	64 (48.5)	68 (51.5)	0.615	1.14 (0.69–1.85)
	**Female**	60 (49.2)	62 (50.8)			63 (51.6)	59 (48.4)		
**Age category**	**≤ 18**	18 (51.4)	17 (48.6)	0.68 0.92	1.16 (0.56–2.37) 1.06 (0.35–3.17)	18 (51.4)	17 (48.6)	0.85 0.92	1.07 (0.52–2.19) 1.06 (0.35–3.17)
	**19–64**	95 (47.7)	104 (52.3)			99 (49.7)	100 (50.3)		
	**≥ 65**	10 (50.0)	10 (50.0)			10 (50.0)	10 (50.0)		
**Occupancy**	**Urban**	47 (34.3)	90 (65.7)	0.001	3.550 (2.11–5.96)	48 (35.0)	89 (65.0)	0.001	3.85 (2.28–6.49)
	**Rural**	76 (65.0)	41 (35.0)			79 (67.5)	38 (32.5)		
**Clinical symptoms**	**Fever**	11 (47.8)	12 (52.2)	0.952	1.03 (0.43–2.42)	11 (47.8)	12 (52.5)	0.827	1.10 (0.46–2.59)
	**Fever & Chills**	19 (35.2)	35 (64.8)	0.030	1.99 (1.07–3.72)	20 (37.0)	34 (63.0)	0.033	1.95 (1.05–3.63)
	**Muscle aches**	30 (41.1)	43 (58.9)	0.139	1.51 (0.87–2.62)	31 (42.5)	42 (57.5)	0.128	1.53 (0.88–2.64)
	**Headache**	31 (44.9)	38 (55.1)	0.496	1.21 (0.7–2.11)	23 (47.8)	36 (52.5)	0.672	1.12 (0.64–1.96)
	**Sore throat**	25 (38.5)	40 (61.5)	0.064	1.72 (0.97–3.06)	25 (38.5)	40 (61.5)	0.032	1.87 (1.05–3.33)
	**Cough**	20 (38.5)	32 (61.5)	0.109	1.67 (0.89–3.1)	21 (40.4)	31 (59.6)	0.122	1.63 (0.87–3.02)
	**Loss of smell & taste**	6 (28.6)	15 (71.4)	0.065	2.52 (0.94–6.72)	6 (28.6)	15 (71.4)	0.047	2.70 (1.01–7.20)
	**Diarrhea & Vomiting**	1 (20.0)	4 (80.0)	0.232	3.84 (0.42–34.86)	1 (20.0)	4 (80.0)	0.210	4.09 (0.45–37.17)
	**Shortness of breath**	4 (14.8)	23 (85.2)	0.001	6.33 (2.12–18.9)	7 (25.9)	20 (74.1)	0.011	3.20 (1.30–7.87)
	**Other**	14 (45.2)	17 (54.8)	0.698	1.16 (0.54–2.47)	14 (45.2)	17 (54.8)	0.566	1.24 (0.58–2.65)
**Underlying health conditions (UHC)**	**Endocrine diseases**	2 (18.2)	9 (81.8)	0.059	4.46 (0.95–21.08)	2 (18.2)	9 (81.8)	0.049	4.76 (1.00–22.52)
	**Liver diseases**	1 (50.0)	1 (50.0)	0.964	0.94 (0.06–15.17)	1 (50.0)	1 (50.0)	0.99	1 (0.06–16.16)
	**Kidney diseases**	1 (20.0)	4 (80.0)	0.232	3.84 (0.42–34.86)	1 (20.0)	4 (80.0)	0.21	4.09 (0.45–37.17)
	**Gastrointestinal diseases**	1 (50.0)	1 (50.0)	0.964	0.94 (0.06–15.17)	1 (50.0)	1 (50.0)	1.000	1.00 (0.06–16.16)
	**Pulmonary diseases**	0 (0.0)	4 (100)	0.232	3.84 (0.42–34.86)	0 (0.0)	4 (100.0)	0.21	4.09 (0.45–37.2)
	**Cardiovascular diseases**	0 (0.0)	7 (100)	0.073	6.89 (0.84–56.82)	0 (0.0)	7 (100.0)	0.064	7.35 (0.89–60.63)
	**No UHC**	117(51.5)	110 (48.5)	0.006	3.72 (1.45–9.56)	121 (53.3)	106 (46.7)	0.004	4 (1.55–10.27)

### Statistical agreement between the results of assays and LICT

Statistical analysis of data using the Chi-square test showed that there was a statistically significant relationship between the LICT and SARS-CoV-2 infection using the TaqMan One-Step RT-qPCR test result (*P* < 0.001). So, 28 and 24 patients with a positive One-Step RT-qPCR and One-Step LAMP test for SARS-CoV-2 respectively also showed LICT. None of the study participants with a negative result for the One-Step RT-qPCR assay had a positive LICT. According to the Kappa coefficient, the agreement between the result of the TaqMan One-Step RT-qPCR test with LICT was 32% which according to *P* < 0.001, this agreement was statistically significant (the accuracy of the result of the tests was 57%) (Table 5).

**Table 5:** The consensus of the results of TaqMan One-Step RT-qPCR, One-Step LAMP, and LICT in the diagnosis of SARS-CoV-2 among patients

		LICT		Total	
		Negative n (%)	Positive n (%)		
**One-Step LAMP**	**Negative**	**123 (48.4)**	**4 (1.6)**	**127 (50.0)**	***P*** **< 0.001**
	**Positive**	**103 (40.6)**	**24 (9.4)**	**127 (50.0)**	**Kappa = 24%**
**One-Step RT-qPCR**	**Negative**	**123 (48.4)**	**0 (0.0)**	**123 (48.4)**	***P*** **< 0.001**
	**Positive**	**103 (40.6)**	**28 (11.0)**	**131 (51.6)**	**Kappa = 32%**

## Discussion

The recent rapid epidemic expansion of SARS-CoV-2 has become a major threat to the world [2, 3]. Rapid, accurate, and specific diagnosis of SARS-CoV-2 infection, is very helpful and vital in controlling the disease epidemic and timely treatment among patients especially in poor or undeveloped countries [24, 25]. In the present study, a rapid One-Step LAMP method was developed and compared to the TaqMan One-Step RT-qPCR assay for the sensitive and specific detection of SARS-CoV-2 in an NP swab sample of patients suspected of COVID-19 living in deprived western areas of Iran. Based on the Cohen’s kappa coefficient (κ) test, the agreement rate between the two tests was 97% (51.6 and 50% positivity rate respectively), which considering *P* < 0.001 was statistically significant. This finding shows the accuracy and reliability of the results obtained from the One-Step LAMP technique compared to the One-Step RT-qPCR assay. The LAMP technology has been utilized successfully to detect and identify SARS-CoV-2 infection in clinical samples of patients [10, 11, 14, 22, 24–37]. In the current survey, the One-Step LAMP assay detected the SARS-CoV-2 at 1 × 10^1^ copies of the RNA standard strain, representing a high sensitivity of this technique (Fig. 4A and B). Compared to quantitative PCR, one of the remarkable advantages of the LAMP technique is the relatively high reaction speed and review of test results in 15 to 60 minutes [14, 22]. The amplification reaction accelerators which reduce the reaction time required for LAMP are the extra two forward and backward loop primers (LF and LB primers) used in the assay [39]. Wei and colleagues also successfully developed an RT-LAMP assay to directly detect SARS-CoV-2 in clinical NP swab samples without the need for prior RNA extraction [19]. In addition to the high reaction speed, the specificity of the LAMP technique has also been reported to be very high in most studies, so false-positive results are rarely reported in this method [13–15]. Similar to this in the present study, the positive results observed in all positive controls and a negative result in all samples with non-SARS-CoV-2 templates represent 100% specificity of the One-Step LAMP assay. The reason for the high specificity of the LAMP technique is several primers (4–6 primers) that recognize different sequences (6–8 regions) of target DNA [14, 22, 23]. Zhu et al. developed a multiplex reverse transcription loop-mediated isothermal amplification (mRT-LAMP) coupled with a nanoparticle-based lateral flow biosensor (LFB) assay with 100% analytical sensitivity and specificity for diagnosing COVID-19 [24]. Likewise, Fowler et al. described a new rapid SARS-CoV-2 RT-LAMP assay for use on extracted RNA or directly from NP swab samples of patients with an overall diagnostic sensitivity and specificity of 97 and 99% respectively [22]. Cost-effectiveness, simplicity, no need for expensive laboratory equipment, and usability in field conditions are other reported advantages of the LAMP technique [10, 11, 14]. Although SARS-CoV-2 can be detected using RT-PCR, the technique has limitations such as inadequate access to reagents and equipment, possibilities limiting the level of biosafety, and technical complexity. In addition, this method is associated with a high rate of false-negative results, mainly due to the non-standard collection of respiratory samples [22, 24, 39].

As mentioned, the NP swab was used in this study. NP samples are routinely used for RT-qPCR. The NP swab collection process’s major flaws are invasive and sometimes not feasible for noncooperative patients [40]. Some patients coming to clinical facilities for COVID-19 testing often sneeze due to irritation inside the nostrils. Droplet or aerosol generation through such a process may infect the healthcare staff [41]. For this reason, suitable alternatives such as saliva and the anterior nasal swab can be used. A notable advantage of the detection of COVID-19 through RT-PCR of saliva is that it reduces the chances of spreading SARS-CoV-2. A study evaluated saliva and NP samples, and the sensitivity was found to be 85.2% for saliva and 94.5% for NP [42]. Another study has reported the presence of SARS-CoV-2 in the nasal vestibule and the sensitivity of testing this particular specimen for diagnosis of SARS-CoV-2 infection. This study points out that Nasal vestibule swab sampling is a promising alternative for the detection of SARS-CoV-2 infection, as this collection site is more accessible and more comfortable for the patient compared to the oropharynx [43].

In the current study, no significant relationship was observed between the variables of gender, age group, and infection with SARS-CoV-2. In other words, SARS-CoV-2 infection can be seen in both genders and any age group. Similar to these results in the study by Novosad et al. no statistically significant relationship was found between SARS-CoV-2 infection and gender and age group [44]. While in the study by Xu et al., the rate of SARS-CoV-2 infection among males and the 30–39 and 40–49 age groups was higher [45]. On the other hand, a statistically significant relationship was found between residency, having chronic UHC, and infection with SARS-CoV-2 (*P* < 0.001). Most patients with a positive test result for SARS-CoV-2 infection lived in the urban area (65.7%) and had at least one chronic UHC (77.8%). According to the univariate logistic regression test, the chance of infection with the Covid-19 virus using the TaqMan One-Step RT-qPCR test in people resident in urban areas was 3.85 times (CI = 2.28–6.49) higher than in rural resident’s people. Research has shown the impact of population and travel as well as travel restrictions on the SARS-CoV-2 epidemic worldwide [46]. Moreover, based on the univariate logistic regression test, the chance of being infected with the SARS-CoV-2 was approximately 4 times higher (CI = 1.55–10.26) in patients with chronic UHC than in people without UHC. The effects of UHC on COVID-19 mortality were evaluated through a cross-sectional study in India and England. According to their results, the highest mortality rate is related to uncontrolled diabetes and chronic respiratory disease, and the lowest rate is obesity, cancer, and chronic heart disease [47].

Statistical analysis of the results using various statistical tests showed that there is a statistically significant relationship between SARS-CoV-2 infection and some clinical symptoms (fever and chills, and shortness of breath), while such a relationship was not found between other clinical symptoms (fever, headache, muscle aches, cough, diarrhea, and vomiting, sore throat, and loss of taste and smell) and SARS-CoV-2 infection. In a similar study by Sarker et al., patients’ most frequently reported symptoms were fever/pyrexia, cough, body ache/pain, fatigue, and headache respectively [47]. Numerous factors, including age, sex, race, and immune status appear to play a role in the development of clinical symptoms during SARS-CoV-2 infection (Table 4).

The LICT was observed in 11.0% of patients with a positive test for SARS-CoV-2 infection while none of the study participants with a negative result for the RT-qPCR assay had a positive LICT. The agreement between the result of the TaqMan One-Step RT-qPCR test with LICT was 32% which according to *P* < 0.001, this agreement was statistically significant (Table 5). This finding indicates a relative agreement between the results of molecular tests and the results of CT scans of the lungs in patients suspected of SARS-CoV-2 infection, which confirms the use of molecular tests along with CT scans of the lungs is very helpful and effective to ensure that people are infected by SARS-CoV-2 infection. Since the beginning of the outbreak of this disease, CT scanning of the lung has emerged as a promising way of research and care. Especially at the beginning of the outbreak, CT scans attracted the medical community’s attention. Recent research shows that the COVID-19 diagnosis from CT scans and X-rays can lower the burden on short supplies of RT-PCR test kits [48, 49].

## Conclusions

In conclusion, the results showed that compared to TaqMan One-Step RT-qPCR, One-Step LAMP is a fast and efficient technique for detecting SARS-CoV-2 among suspected individuals living in poor or undeveloped countries due to its simplicity, low cost, sensitivity, and specificity. Consistency of One-Step LAMP test results with TaqMan One-Step RT-qPCR and imaging findings in patients’ lung CT scans also confirms the accuracy and reliability of the One-Step LAMP technique for the diagnosis of SARS-CoV-2 infection in suspected patients. Therefore, it has great potential as a useful diagnostic tool for disease epidemic control, timely treatment, and general health protection, especially in poor or undeveloped countries.

## Supplementary Information

**Additional file 1.**

**Additional file 2.**

## Data Availability

All data generated or analyzed during this study are included in this published article.

## Abbreviations

NP: Nasopharyngeal
LAMP: Loop-mediated isothermal amplification
SARS-CoV-2: Severe Acute Respiratory Syndrome Coronavirus 2
FIP: Forward internal primer
BIP: Backward internal primer
VTM: Viral transport media
RdRp: RNA-dependent RNA polymerase
dNTP: Deoxynucleoside triphosphates
LICT: Lung involvement in computed tomography
