# Supplementary figures and images for: Rapid, sensitive, and specific detection of SARS-CoV-2 in nasopharyngeal swab samples of suspected patients using a novel one-step loop-mediated isothermal amplification (one-step LAMP) technique
Source: BMC Microbiol. 2023 Mar 7;23:63. doi: 10.1186/s12866-023-02806-z (PMC9989590; doi:10.1186/s12866-023-02806-z)

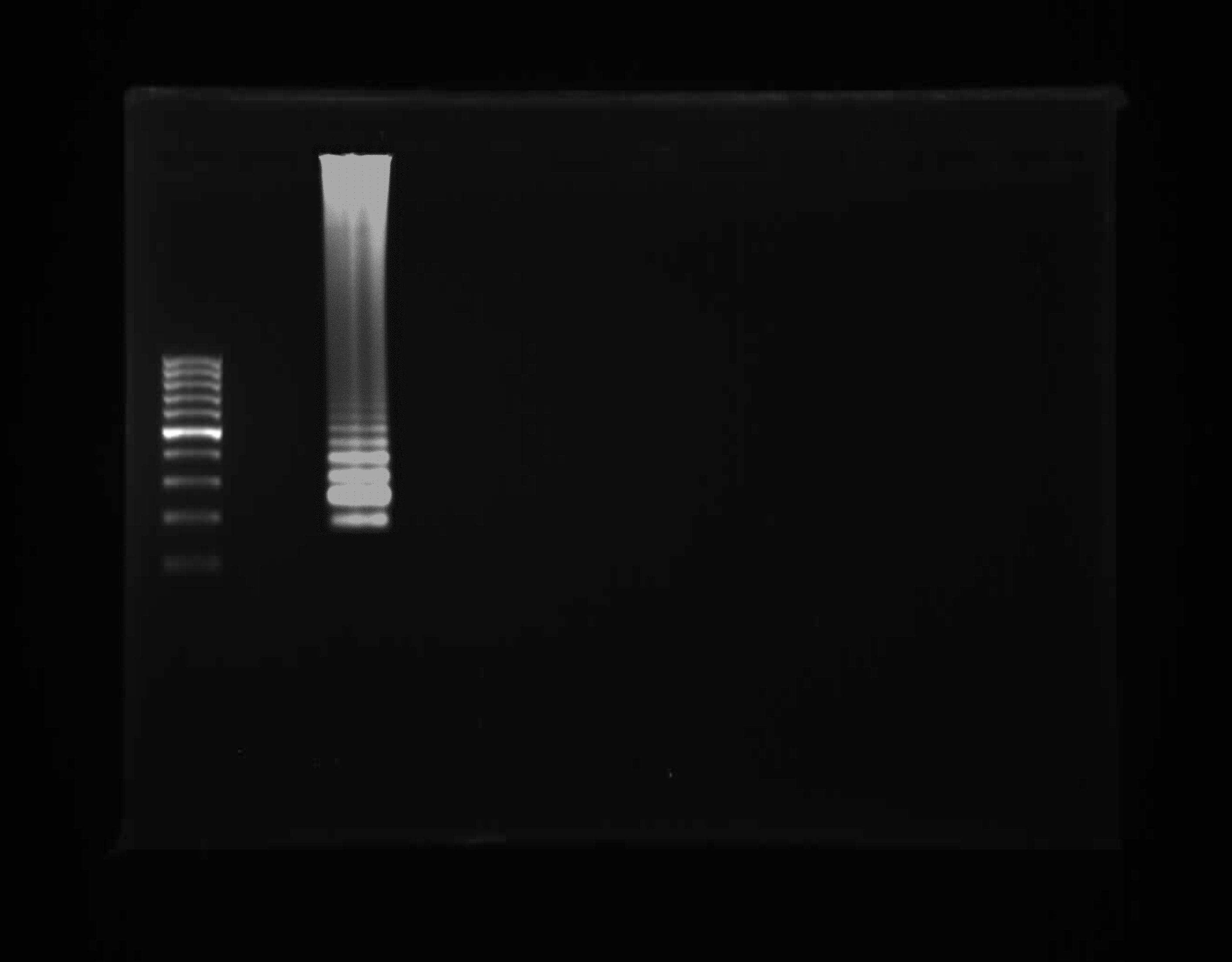

Supplement: Supplementary file 1 — Additional file 1. [file 12866_2023_2806_MOESM1_ESM.jpg]

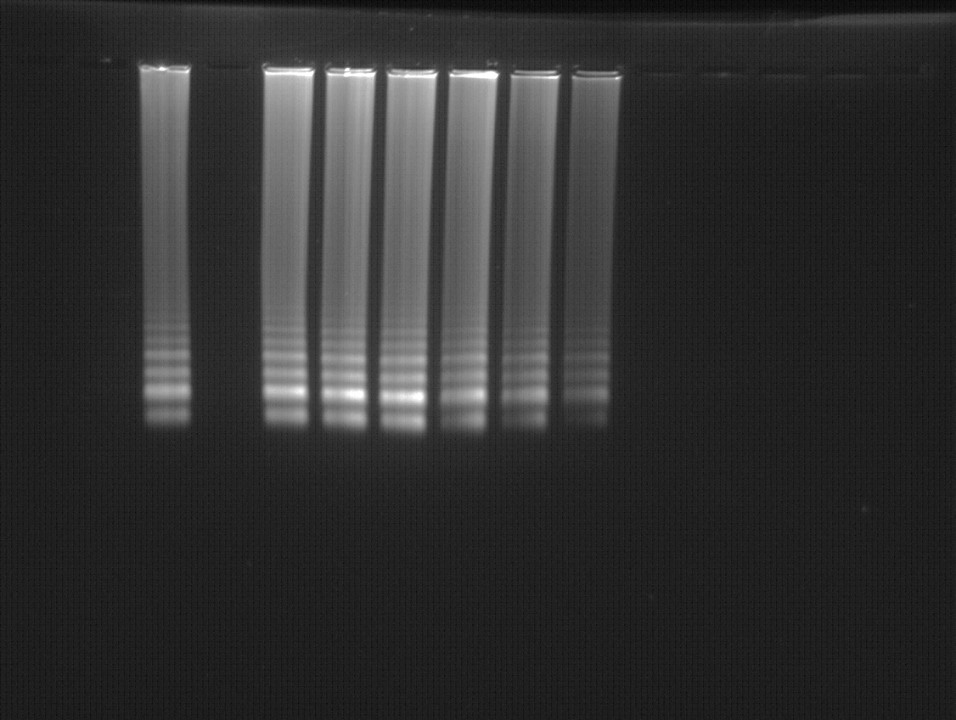

Supplement: Supplementary file 2 — Additional file 2. [file 12866_2023_2806_MOESM2_ESM.jpg]
